# Loneliness to consumption intention: emotional bonding and escapism in AI emotional companion app use

**DOI:** 10.3389/fpubh.2026.1964252

**Published:** 2026-10-02

**Authors:** Pei Yao, Zirong Tian, Jongmoo Kim

**Affiliations:** 1 College of Media and Arts (School of International Education), Tianjin University of Sport, Tianjin, China; 2 School of Media and Communications, Dankook University, Yongin-si, Gyeonggi-do, Republic of Korea

**Keywords:** AI emotional companion app, loneliness, actual-ideal self-discrepancy, emotional bond with the AI, experience-enhancing consumption intention

## Abstract

With the diffusion of generative AI, AI emotional companion apps have emerged as a new domain of digital consumption in which users may pay to extend relationship-like experiences. This study examines how loneliness and actual-ideal self-discrepancy are associated with experience-enhancing consumption intention and continuance intention through two competing pathways: emotional bonding with the AI (relational compensation) and escapism (avoidant compensation). The emotional bond with the AI is modeled as a second-order construct comprising anthropomorphism, emotional responsiveness, and parasocial intimacy. Data were collected from 393 adult users in China who used AI emotional companion apps at least twice per week and were analyzed using structural equation modeling. Loneliness and actual-ideal self-discrepancy were positively associated with both emotional bonding and escapism. However, significant indirect associations with experience-enhancing consumption intention and continuance intention emerged only through the emotional-bond pathway. Direct comparisons further showed that the relational indirect effects were significantly stronger than the avoidant indirect effects across all antecedent–outcome combinations. Escapism was significantly associated with both outcomes when modeled alone but became nonsignificant when emotional bonding was included, suggesting that its association with consumer responses largely overlaps with the relational pathway. A partial-mediation analysis further showed that actual-ideal self-discrepancy retained a significant direct association with continuance intention. These findings suggest that relational compensation, rather than avoidant compensation, is more closely associated with willingness to pay for experience enhancement and intention to continue using AI companions. The study extends compensatory-use, self-discrepancy, and parasocial-interaction perspectives to the AI-companion context by linking psychological vulnerability to experience-enhancing consumption intention and continuance intention, while highlighting implications for responsible relational design and user protection.

## 1 Introduction

Paying for interaction with an AI character has become an increasingly visible form of digital consumption. In China, the AI emotional companion industry is projected to grow from RMB 1.211 billion in 2024 to RMB 3.866 billion in 2025 and to exceed RMB 59.5 billion by 2028 (1). Platforms such as Xingye (星野), Maoxiang (猫箱), and Glow monetize interaction through premium dialogue, voice services, character customization, exclusive content, and subscriptions. Unlike conventional subscription software, these services increasingly monetize the relational experience itself: users pay not simply for additional functions but for features that may sustain or enrich relationship-like interaction with an AI character. This emerging form of relational consumption raises an important question about why some users report stronger willingness to invest in and continue such emotionally meaningful AI interactions.

The present study does not measure actual expenditure or repeated payment behavior. Instead, it focuses on users’ self-reported willingness to pay for features that may enrich their relational experience with an AI character. Experience-enhancing consumption intention should therefore be understood as a consumption intention rather than actual transaction behavior. This distinction is consistent with recent research showing that AI companion platforms increasingly monetize access to relationship-like capacities through subscription and premium-service architectures (2).

This form of relational monetization is particularly relevant when users turn to AI companions for emotional support. AI emotional companion apps provide interaction that is continuously available, emotionally responsive, and nonjudgmental, potentially offering compensation for unmet relational needs. At the same time, recent Chinese regulation of anthropomorphic AI interaction has highlighted concerns including emotional dependence, emotional manipulation, excessive immersion, and user protection (3). These developments make it important to examine relational monetization together with users’ psychological characteristics and responsible platform design.

Despite the rapid growth of AI companion use, less is known about why users with different psychological characteristics report stronger willingness to pay for enriched AI-companion experiences and stronger continuance intention. Prior research has focused largely on technology-acceptance factors, such as usefulness, human-likeness, and response quality, or on whether AI companions may alleviate loneliness (4). Comparatively less attention has been paid to the psychological pathways through which loneliness and self-related difficulties are associated with emotional bonding, escapism, consumption intention, and continued use.

The present study addresses this gap by focusing on loneliness and actual-ideal self-discrepancy as two theoretically specified forms of psychological vulnerability. Loneliness reflects unmet needs for social and emotional connection (5), whereas actual-ideal self-discrepancy refers to the perceived gap between one’s actual and ideal self (6); both may be associated with compensatory consumption (7). Here, psychological vulnerability is used only as a theoretical shorthand for higher self-reported loneliness and actual-ideal self-discrepancy and does not imply a clinical diagnosis or a clinically vulnerable population. The study distinguishes two compensatory routes: emotional bonding with the AI as relational compensation and escapism as avoidant compensation. Drawing on mood-management and compensatory-use perspectives (8, 9), self-discrepancy theory (6), uses and gratifications theory (10), and parasocial interaction theory (11), the competing-mediation model examines how loneliness and actual-ideal self-discrepancy are associated with experience-enhancing consumption intention and continuance intention through these two pathways. Because the data are cross-sectional, the proposed ordering is theoretically specified rather than evidence of temporal or causal sequence, and reciprocal relationships remain possible.

This study makes three main contributions. First, it extends research beyond technology-acceptance explanations by examining loneliness and actual-ideal self-discrepancy as psychological correlates of AI-companion consumption-related and continuance intentions. Second, it conceptualizes the emotional bond with the AI as a higher-order construct comprising anthropomorphism, emotional responsiveness, and parasocial intimacy, thereby shifting attention from functional satisfaction to relationship-like bonding. Third, by directly comparing emotional bonding and escapism within a single model, it clarifies whether consumer responses are more closely associated with relational compensation or avoidant immersion. From a public mental health perspective, the study does not directly assess mental health outcomes or problematic AI use. Rather, it identifies how higher self-reported loneliness and actual-ideal self-discrepancy are associated with relational engagement and consumption- and continuance-related intentions, providing evidence relevant to prevention-oriented user protection and responsible AI-companion design.

## 2 Theoretical background

AI emotional companion apps provide a distinctive context for examining compensatory engagement because repeated, personalized, and emotionally responsive interaction can support both relationship-like bonding and avoidant immersion. Building on compensation theory, self-discrepancy theory, uses and gratifications theory, and parasocial interaction theory, the following sections develop the theoretical basis for the study’s two competing pathways: emotional bonding with the AI as relational compensation and escapism as avoidant compensation.

### 2.1 Core theoretical framework

#### 2.1.1 Compensation theory and the compensatory-use perspective

Compensation theory explains how individuals respond to psychological deficits by seeking alternative resources to restore psychological balance (7, 12). In media contexts, the compensatory-use perspective similarly suggests that users may turn to digital environments to cope with unmet relational or self-related needs (9, 13, 14). Accordingly, loneliness and actual-ideal self**-**discrepancy may function as psychological vulnerabilities that motivate compensatory engagement with AI emotional companion apps.

In this context, compensation may take two distinct forms. Escapism represents an avoidant route through which users seek temporary relief from real-world discomfort, whereas emotional bonding with the AI represents a relational route in which the AI becomes a target of attachment through repeated, responsive interaction (15, 16). Because compensatory consumption can involve spending aimed at offsetting psychological deficits or sustaining desired experiences (7, 17), willingness to pay for premium or experience-enhancing AI features may be associated with these compensatory processes. The present study therefore compares the relational and avoidant routes in relation to experience-enhancing consumption intention and continuance intention.

#### 2.1.2 Self-discrepancy theory

Self-discrepancy theory explains the affective consequences of perceived gaps within the self-concept (18). This study focuses on actual-ideal self-discrepancy, defined as the perceived gap between one’s actual attributes and the person one ideally wishes to be. Such discrepancies are associated with disappointment, dejection, and loneliness and may motivate compensatory responses aimed at reducing the discomfort created by the self-gap (7, 19, 20). Accordingly, actual-ideal self-discrepancy is treated as an important psychological vulnerability in the present study.

Digital environments may provide a particularly accessible context for such compensation because they allow greater control over self-presentation and opportunities for ideal-self projection (21). Prior research shows that users may project idealized selves onto virtual characters and form emotional bonds with them, while self-discrepancy has also been linked to compensatory consumption aimed at repairing or expressing an ideal identity (22–25). In AI emotional companion apps, personalized interaction and experience-enhancing features may therefore provide both relational and consumption-related channels through which users respond to actual-ideal self-discrepancy.

#### 2.1.3 Uses and gratifications theory

Uses and gratifications theory views media users as active agents who select media to satisfy psychological and social needs (10). This perspective has been widely applied to digital and AI-mediated services to explain why users engage with particular platforms and interaction formats (26–28). In AI emotional companion apps, users may seek emotional support, companionship, social connection, and opportunities for self-exploration, making these services a potential source of both emotional and relational gratification (4, 15, 16).

From this perspective, gratifying AI interactions may be associated with deeper engagement and downstream consumer responses. Prior research has linked media gratification and immersion to continuance and purchase-related intentions, while emotionally rewarding AI interactions may strengthen attachment and continued use (29–31). Accordingly, users who derive greater emotional or relational gratification from AI companions may also report stronger willingness to pay for experience-enhancing features and stronger continuance intention.

#### 2.1.4 Parasocial interaction theory and the emotional bond with AI

Parasocial interaction was first introduced by Horton and Wohl (11) to describe one-sided yet relationship-like interaction between media audiences and media figures. It has since been used to explain audiences’ emotional investment in media figures and its cognitive and behavioral consequences (32, 33). The finding that lonelier audiences rely more heavily on parasocial relationships suggests that such relationships may serve as a channel for compensating unmet relational needs (34).

In digital environments, parasocial interaction has extended beyond traditional media figures to brands, virtual characters, and conversational agents, and has been associated with consumer responses such as loyalty and purchase intention (35, 36). AI emotional companion apps represent a further extension of this process because AI characters can respond to users in real time and in a personalized manner. These interactive features may strengthen perceptions of reciprocity and support the formation of attachment-like or friendship-like bonds with chatbots (16, 37).

#### 2.1.5 Escapism as an avoidant compensation route

Escapism refers to a coping tendency in which attention is diverted to a virtual environment to relieve real-world pressure, negative affect, or loneliness (38). Stenseng et al. (39) conceptualized escapism as including both self-suppression, oriented toward avoiding real-world problems, and self-expansion, oriented toward pursuing positive experiences. Related research on anthropomorphized digital entities and AI chatbots suggests that escapist motives can be associated with continued engagement and problematic use (40, 41). This study therefore positions escapism as the mediator of an avoidant compensation route that is theoretically distinct from relational compensation.

Whereas emotional bonding with the AI is oriented toward relationship formation with the target, escapism is oriented toward temporary relief from real-world tension. The model therefore examines whether this avoidant route is associated with experience-enhancing consumption intention and continuance intention, and whether it retains explanatory power when the relational route is modeled simultaneously. Continuance intention is included as a downstream consumer-response outcome, following information-systems research on continued platform use (42). However, the present study does not test the full expectation-confirmation process because it does not include confirmation or satisfaction. Accordingly, the theoretical focus remains on the comparison between emotional bonding and escapism as competing mediating routes.

### 2.2 Psychological vulnerability: loneliness and actual-ideal self-discrepancy

Loneliness is not simply the state of being alone but a painful subjective experience stemming from the absence of intimate relationships or social support (43). Individuals with insecure attachment tendencies are more vulnerable to chronic loneliness, which is often accompanied by low self-esteem (44); chronic loneliness also weakens authenticity experiences and subjective well-being through rumination (45). The psychological pain stemming from unmet relational needs is associated with a tendency to seek emotional compensation externally. The findings that lonely individuals rely more on parasocial relationships with media figures (34) and more strongly perceive nonhuman targets as humanlike (46) indicate that loneliness can be an antecedent of alternative relationship formation.

Actual-ideal self-discrepancy, the perceived gap between the actual and ideal selves, is a key antecedent of psychological maladjustment such as depression and social anxiety (20, 47). In an environment saturated by social media, perception of this gap can be amplified by appearance anxiety and continual social comparison with peers (48, 49). The two vulnerabilities are not unrelated. Frustration of the self-concept in reality can deepen loneliness by way of interpersonal avoidance (48), and the rumination that accompanies loneliness can intensify negative self-evaluation and heighten tendencies toward inauthentic self-presentation online (45, 49). When social resources to buffer such deficits are lacking in reality (50), individuals may divert their attention to virtual environments. Digital spaces, high in anonymity and controllability, relax the constraints of self-expression and offer opportunities for identity reconstruction (21, 51), and projecting the ideal self onto a digital target such as an AI emotional companion can be one concrete form this takes. On this basis, the study examines loneliness and actual-ideal self-discrepancy as theoretically specified correlates of the two compensation routes in the context of AI emotional companion app use.

### 2.3 Emotional bond with the AI (BOND): an integrative construct of anthropomorphism, emotional responsiveness, and parasocial intimacy

The emotional bond with the AI refers to the extent to which users perceive an AI emotional companion not merely as a technical tool but as a relational target that is humanlike, emotionally responsive, and capable of providing intimacy. This study conceptualizes this bond as an integrative experience composed of three facets: anthropomorphism, emotional responsiveness, and parasocial intimacy. Together, these facets capture the process through which human-AI interaction may shift from instrumental use to a relationship-like experience (37, 52).

Anthropomorphism refers to the attribution of human characteristics, motives, and emotions to a nonhuman target (53). In AI emotional companion apps, anthropomorphism may arise not only from visual or character cues but also from conversational naturalness, affective language, memory-based personalization, and a consistent character narrative. Prior research has shown that anthropomorphic perceptions of AI agents and chatbots are associated with trust, psychological closeness, engagement, and other positive consumer responses (54–56), suggesting that perceiving the AI as humanlike is a basic condition for relationship-like interaction.

Emotional responsiveness further deepens this relational perception. In intimacy research, perceived partner responsiveness refers to the extent to which one feels understood, validated, and cared for by another (57). In AI-mediated interaction, emotionally expressive and supportive responses can shape users’ perceptions of support and their evaluations of the interaction (58, 59). Related research further suggests that AI can be experienced as a socially meaningful interaction partner, including as a mirror, mate, mentor, or socially present collaborator (60, 61).

As anthropomorphism and emotional responsiveness accumulate through repeated interaction, users may develop parasocial intimacy with the AI. This refers to a sense of intimate, relationship-like connection with a media or virtual target, extending the tradition of parasocial relationship research to the AI-companion context (11, 34). Prior studies have shown that users can experience chatbots as friends or attachment figures (16, 62), and that emotionally capable, anthropomorphic AI can evoke strong attachment (37). On this basis, the present study treats the emotional bond with the AI as a second-order construct comprising anthropomorphism, emotional responsiveness, and parasocial intimacy, and positions it as the key mediator of the relational compensation route linking psychological vulnerability to experience-enhancing consumption intention and continuance intention.

### 2.4 Consumer responses: experience-enhancing consumption intention and continuance intention

In digital environments, consumption is increasingly driven not only by utilitarian value but also by hedonic and experiential value (63, 64). Across digital and experiential consumption contexts, hedonic experience, social presence, and brand experience have been associated with engagement, purchase intention, and emotional attachment (59, 65–67). In AI emotional companion apps, paid behaviors such as purchasing virtual outfits, unlocking exclusive voices, or sending gifts can be understood as attempts to enrich a relational experience with an AI character. The present study conceptualizes this outcome as experience-enhancing consumption intention, referring to users’ self-reported willingness to pay for features that may enhance their interactional and relational experience with the AI, rather than actual expenditure or impulse buying.

Continuance intention is included as a second downstream consumer-response outcome. Prior information-systems and media-platform research has treated continuance intention as a key indicator of user retention and has shown that continued use is associated with hedonic value, perceived enjoyment, satisfaction, and affective value derived from system interaction (68–70). Across human-computer interaction and digital-platform research, anthropomorphism and social presence have been linked to continuance intention (71–73).

In the relational context of AI emotional companion apps, experience-enhancing consumption intention and continuance intention may be closely tied to emotional bonding. In chatbot contexts, parasocial relationships have been associated with continuance intention (74). More broadly, perceived value, satisfaction, and relational experience have also been linked to continued use across digital services (75–78). However, these outcomes may also be associated with avoidant immersion. The present study therefore compares whether relational bonding or escapism is more strongly associated with experience-enhancing consumption intention and continuance intention in the competing-mediation model.

### 2.5 Research gap and theoretical positioning

Prior research relevant to AI companions and adjacent virtual-agent contexts has largely developed along three lines: technology-adoption and continuance studies emphasizing anthropomorphism, perceived value, and continued use (28, 55, 56, 71, 75); escapism-oriented work examining digital environments as spaces for relief from real-world stress (9, 13, 14, 38–41); and relational studies examining friendship-like, parasocial, or attachment-like bonds with chatbots and AI companions (16, 30, 37, 60, 62). What remains less clear is how psychological vulnerability is associated, within a single model, with relational and avoidant compensatory experiences and with downstream consumer responses. The present study addresses this gap by taking loneliness and actual-ideal self-discrepancy as common antecedents and comparing two theoretically specified pathways: emotional bonding with the AI as a relational compensation route and escapism as an avoidant compensation route. By testing their relative associations with experience-enhancing consumption intention and continuance intention, the study clarifies whether AI-companion use among individuals reporting higher levels of loneliness or actual-ideal self-discrepancy is more closely tied to relationship-like bonding or to avoidant immersion.

## 3 Method

### 3.1 Research model and hypotheses

This study examines the theoretically specified pathways through which psychological vulnerability is associated with consumer responses in AI emotional companion app use. Drawing on the compensatory-use perspective and compensatory consumption research, loneliness and actual-ideal self-discrepancy are treated as psychological deficits that may be associated with media use, relational compensation, and consumption-related intentions (7, 9, 34). The model distinguishes two compensatory routes: a relational compensation route, represented by the emotional bond with the AI, and an avoidant compensation route, represented by escapism.

The central proposition is that, although both routes may be associated with psychological vulnerability, they differ in their associations with downstream consumer responses. Emotional bonding with the AI may be more strongly associated with experience-enhancing consumption intention and continuance intention because it reflects a relationship-like attachment to the AI, whereas escapism may mainly provide temporary relief from real-world stress or self-related discomfort. The study therefore specifies eight direct-effect hypotheses (H1a–H4b) and eight indirect-effect subhypotheses (H5a1–H5d2). The research model is shown in Figure 1.

**Figure 1:**
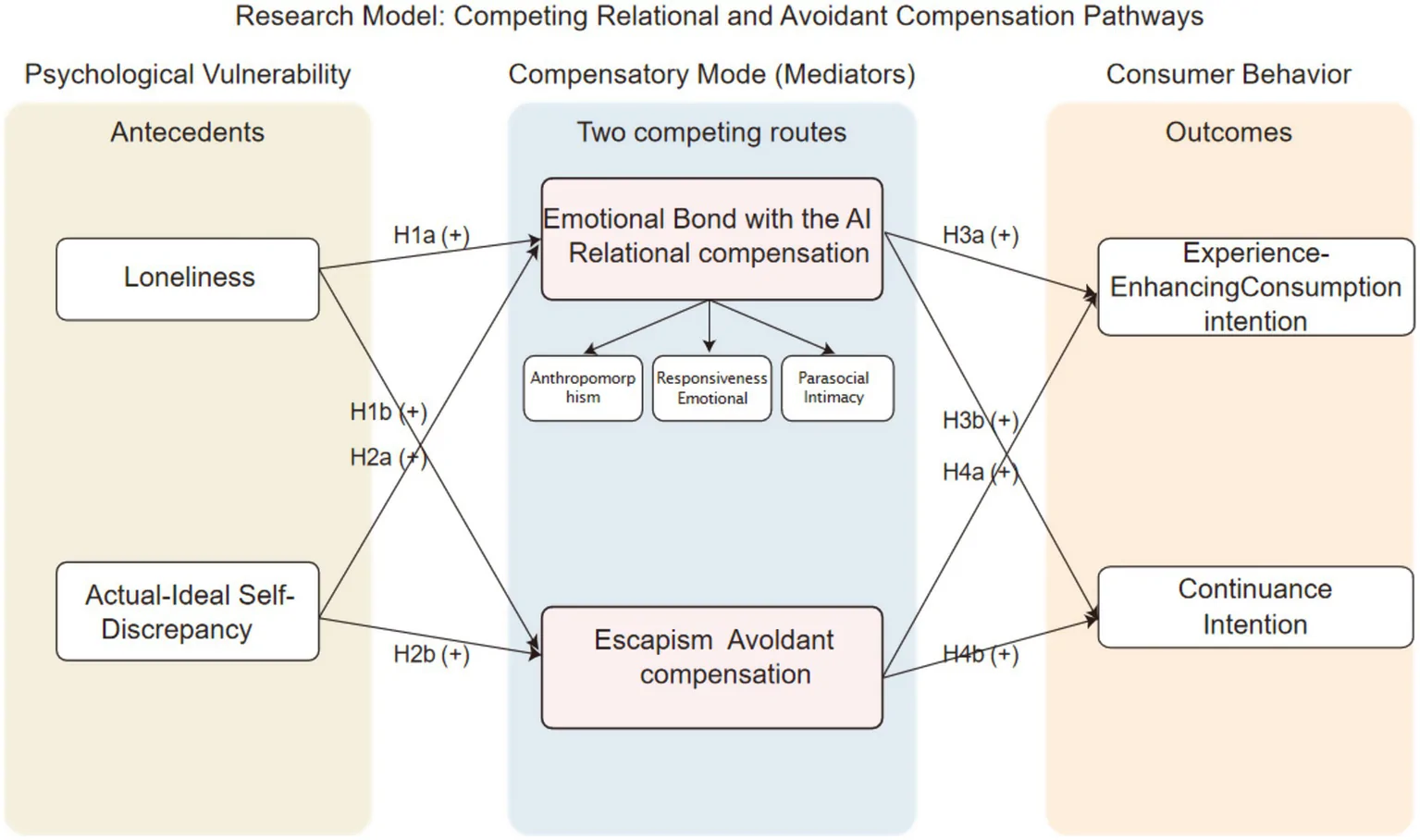
Research model: competing relational and avoidant compensation pathways. Indirect (mediating) effects: emotional bond with the AI (H5a1–H5b2) and escapism (H5c1–H5d2).

#### 3.1.1 Psychological vulnerability and the two compensation routes

The study distinguishes psychological vulnerability into two dimensions: loneliness and actual-ideal self-discrepancy. Loneliness refers to the sense of deficiency an individual perceives in social and emotional relationships (5), and actual-ideal self-discrepancy to the inconsistency between one’s actual self and the self one ideally wishes to be (6). Both deficits may be associated with unmet psychological needs and a tendency to seek alternative resources to offset them. By providing an interlocutor designed to respond consistently and nonjudgmentally, AI emotional companion apps can become potential sources of compensation for these unmet needs.

Lonely individuals tend to form parasocial relationships with media figures or virtual targets to offset an unmet need for social connection (11, 34). Because an AI emotional companion responds consistently to users’ utterances and provides emotional support, higher levels of loneliness may be associated with greater acceptance of the AI as a humanlike relational target and greater perceived emotional closeness. Loneliness may thus be positively associated with anthropomorphism and perceptions of intimacy toward the AI and, in this respect, with stronger emotional bonding with the AI.

*H1a*. Loneliness will be positively associated with the emotional bond with the AI.

Individuals with greater actual-ideal self-discrepancy may be motivated to seek opportunities in alternative spaces to express or experience aspects of their ideal self (6). AI emotional companion apps allow users to construct an ideal relational experience through an interlocutor that responds in the manner they desire, and in this process users come to perceive the AI as an intimate presence aligned with their emotional needs. Greater actual-ideal self-discrepancy was therefore expected to be positively associated with the emotional bond with the AI.

*H1b*. Actual-ideal self-discrepancy will be positively associated with the emotional bond with the AI.

A psychological deficit, however, can also be compensated in a manner different from relationship formation. According to the compensatory-use perspective, individuals in a negative affective state may use media as a means of escaping real-world stress or deficiency, and this avoidant motive is conceptualized as escapism (9, 39). Because the uncomfortable affect associated with loneliness and actual-ideal self-discrepancy may also be associated with a desire for temporary withdrawal from reality, the two vulnerabilities are expected to be positively associated with escapist use of AI emotional companion apps.

*H2a*. Loneliness will be positively associated with escapism.

*H2b*. Actual-ideal self-discrepancy will be positively associated with escapism.

#### 3.1.2 The relational compensation route: emotional bond with the AI and consumer responses

The emotional bond with the AI denotes the extent to which a user perceives an AI emotional companion not merely as a technical tool but as a relational target that is humanlike, emotionally responsive, and capable of providing intimacy. It is a multidimensional relational experience that extends three concepts to the AI-companion context: anthropomorphism (perceiving the target as humanlike; 53), emotional responsiveness (perceiving the AI as appropriately responsive to one’s emotions; 57), and parasocial intimacy (experiencing a sense of intimate relationship; 10).

A stronger emotional bond with the AI may be associated with greater willingness to invest in enriching the relational experience. Because such bonding reflects immersion in and commitment to the relational target, it may be positively associated with experience-enhancing consumption intention, including willingness to pay for premium features, personalization services, and exclusive content.

*H3a*. The emotional bond with the AI will be positively associated with experience-enhancing consumption intention.

The emotional bond may also be an important correlate of continuance intention toward the platform. Users who are emotionally bonded with the AI may repeatedly use the app out of a motive to maintain that relationship. In this sense, continuance intention reflects not only general platform retention but also the user’s willingness to sustain an ongoing relational experience with the AI. The emotional bond was therefore expected to be positively associated with continuance intention.

*H3b*. The emotional bond with the AI will be positively associated with continuance intention.

#### 3.1.3 The avoidant compensation route: escapism and consumer responses

Escapism refers to an avoidant use tendency in which AI emotional companion apps are used to escape real-world stress, anxiety, and loneliness (39). Unlike the emotional bond, which is oriented toward forming a relationship with the target, it is a compensatory mode whose end is withdrawal from reality itself.

If avoidant use provides a satisfying affective experience, users may report stronger consumption-related responses and continuance intention to reinforce that experience. Insofar as escapist immersion provides immediate solace, users may be motivated to invest in paid features or to use the app repeatedly in order to sustain it. The study accordingly hypothesized that escapism, too, would be positively associated with the two consumer responses. Because avoidant compensation does not entail relational bonding with the target, however, its association with consumer responses was expected to be weaker than that of relational compensation.

*H4a*. Escapism will be positively associated with experience-enhancing consumption intention.

*H4b*. Escapism will be positively associated with continuance intention.

#### 3.1.4 Competing mediation: the relative explanatory power of the two routes

Taken together, the paths from H1 through H4 imply that psychological vulnerability is associated with the two consumer responses by way of the two mediators, emotional bond with the AI and escapism. The study tests the statistical indirect associations corresponding to each theoretically specified route. In the relational compensation route, loneliness and actual-ideal self-discrepancy are indirectly associated with experience-enhancing consumption intention and continuance intention through the emotional bond with the AI; in the avoidant compensation route, the same vulnerabilities are indirectly associated with the two consumer responses through escapism.

*H5a1*. The emotional bond with the AI will statistically mediate the association between loneliness and experience-enhancing consumption intention.

*H5a2*. The emotional bond with the AI will statistically mediate the association between loneliness and continuance intention.

*H5b1*. The emotional bond with the AI will statistically mediate the association between actual-ideal self-discrepancy and experience-enhancing consumption intention.

*H5b2*. The emotional bond with the AI will statistically mediate the association between actual-ideal self-discrepancy and continuance intention.

*H5c1*. Escapism will statistically mediate the association between loneliness and experience-enhancing consumption intention.

*H5c2*. Escapism will statistically mediate the association between loneliness and continuance intention.

*H5d1*. Escapism will statistically mediate the association between actual-ideal self-discrepancy and experience-enhancing consumption intention.

*H5d2*. Escapism will statistically mediate the association between actual-ideal self-discrepancy and continuance intention.

Beyond testing the two mediating routes in parallel, the study establishes a competing-mediation structure that compares their relative explanatory power. The key prediction is that the relational compensation route (emotional bond with the AI) mediates the link from psychological vulnerability to consumer responses more strongly than the avoidant compensation route (escapism). The rationale is that emotional bonding reflects a relationship-like connection that may be more closely associated with sustained investment, whereas escapism primarily reflects temporary relief and may therefore show weaker associations with consumer responses. By comparing how strongly relational and avoidant compensation are associated with consumer responses, the study seeks to determine which pathway shows a stronger association with consumption-related and continuance intentions in AI emotional companion app use.

### 3.2 Participants and data collection

The survey targeted adult users who regularly use AI emotional companion apps in China. Both domestically developed apps (e.g., Xingye and Maoxiang) and internationally developed apps available to users in China (e.g., Glow, Replika, and Talkie) were represented. Because the study focuses on how emotional bonding with AI and avoidant AI use are associated with consumer responses, the analysis was limited to active users who used these apps repeatedly rather than one-time or trial users.

Data were collected through Tencent Survey, a Chinese online survey platform, over 8 days from May 23 to May 30, 2026. The introduction presented two screening items confirming prior experience with AI emotional companion apps and a usage frequency of at least twice per week. Only respondents who met both conditions proceeded to the main survey. Of the 420 responses collected, 27 were excluded because of excessively short completion times or clearly inattentive or inconsistent response patterns, leaving 393 responses for the final analysis. The final sample size was also considered adequate for the planned multivariate analyses. With 393 participants and 39 retained measurement indicators, the sample provided approximately 10 observations per indicator. In addition, a sensitivity analysis for a structural regression with two predictors, *α* = 0.05, and statistical power of 0.80 indicated that the available sample could detect an effect of approximately f^2^ = 0.025, suggesting sufficient sensitivity to detect small effects. The final analytic dataset contained no missing values; therefore, no imputation or model-based missing-data procedure was required.

The questionnaire included the measurement items for the principal constructs, each construct containing a reverse-coded item (R) to control for response tendencies, along with items capturing respondents’ general characteristics, including gender, age, education, region of residence, relationship status, length of use, weekly frequency of use, paid-purchase experience, and primary purpose of use. The questionnaire combined two types of measures: those adapted from established scales and those substantially contextualized from established measures for the present study. The initial questionnaire was developed in English and subsequently translated into Chinese for administration. Before formal data collection, all items underwent expert review, translation and independent back-translation, as well as a small-scale pretest.

The data collection adhered to research ethics principles. The introduction explained the study’s purpose, the assurance of anonymity, the principle of voluntary participation, and the possibility of withdrawal, and only respondents who consented proceeded. The study collected no personally identifying information such as names, contact details, or addresses, and the data were used solely as anonymous material for statistical analysis. The study was reviewed and approved by the Academic Ethics Committee of Tianjin University of Sport (Approval No. 2026–069; approved on 29 April 2026) before the commencement of data collection. All participants provided informed consent before taking part in the survey.

### 3.3 Operational definitions and measurement development

All constructs were measured using seven-point Likert scales. The questionnaire combined measures adapted from established scales with items developed or substantially contextualized for the specific setting of AI emotional companion app use. Loneliness (LON) was adapted from Russell’s (5) UCLA Loneliness Scale. Anthropomorphism (ANT), emotional responsiveness (ER), and parasocial intimacy (PI) were adapted from Epley et al. (53) and Bartneck et al. (79), Reis et al. (57), and Horton and Wohl (11) and Rubin et al. (34), respectively, and were modeled as the three lower-order dimensions of the second-order emotional bond with the AI (BOND). Escapism (ESC) was adapted from Stenseng et al. (39), and continuance intention (CI) was adapted from Bhattacherjee (42).

Particular attention was given to actual-ideal self-discrepancy (SIG) and experience-enhancing consumption intention (EECI), because these measures required greater item development and contextual adaptation. The conceptual domain of SIG was derived primarily from Higgins’s (6) self-discrepancy theory, with additional reference to Mandel et al. (7) and Hardin and Lakin (80). The items were designed to capture respondents’ perceived gap between their actual self and the person they ideally wished to be, rather than general dissatisfaction, low self-esteem, or negative affect. EECI was developed for the present context with reference to Zeithaml et al. (81) and was defined as users’ self-reported willingness to pay for features that enhance or extend the interactional and relational experience with an AI-companion, rather than actual expenditure or general purchase intention. Accordingly, the items focused on willingness to pay for premium, personalized, or otherwise experience-enhancing features and services.

Before formal data collection, the initial English item pool was reviewed by four academic experts, including two professors and two scholars holding doctoral degrees. The experts evaluated the items in terms of conceptual relevance, clarity, redundancy, and appropriateness for the AI emotional companion context. Their feedback was used to refine the wording and content of the items and to ensure that the measures adequately represented the intended construct domains. This qualitative expert-review procedure was used to assess content validity; no numerical content-validity index was calculated.

After the English questionnaire had been finalized, it was translated into Chinese by a bilingual specialist proficient in English and Chinese. An independent bilingual English-language specialist, who had not been provided with the original English wording, then back-translated the Chinese version into English. The original and back-translated English versions were compared item by item to assess semantic and conceptual equivalence.

Before the main survey, a small-scale pretest involving approximately 50 respondents was conducted to assess item comprehensibility, wording clarity, and questionnaire flow. Each scale or subscale initially included one reverse-coded item. Following item-level diagnostics, the reverse-coded items were excluded before estimation of the final measurement model, resulting in 39 retained items. The complete measurement items and their corresponding sources are provided in Supplementary Table S1.

### 3.4 Analytic strategy

All analyses in this study were conducted using IBM SPSS Statistics 27.0 and IBM SPSS Amos 27.0. SPSS 27.0 was used for data screening, descriptive statistics, reliability analysis (Cronbach’s α and corrected item-total correlations), common-factor exploratory factor analysis, correlation and partial-correlation analyses, and Harman’s single-factor test. Amos 27.0 was used for the first- and second-order confirmatory factor analyses, the structural equation model, the bootstrap estimation of indirect effects, and the CFA-based common latent method-factor analysis. All confirmatory factor analysis and structural equation modeling parameters were estimated using maximum likelihood (ML) estimation. The final analytic dataset contained no missing values; therefore, no imputation, full-information maximum likelihood, or other model-based missing-data procedure was required. Model fit was evaluated using χ^2^/df, the comparative fit index (CFI), the Tucker–Lewis index (TLI), the root mean square error of approximation (RMSEA), and the standardized root mean square residual (SRMR).

The latent structure of the retained measurement items was first evaluated using common-factor exploratory factor analysis with principal axis factoring and Promax rotation. Confirmatory factor analysis was then conducted to assess composite reliability, average variance extracted, discriminant validity, and the second-order structure of the emotional bond with the AI. Because the primary exploratory, confirmatory, and structural analyses used the full sample, an additional split-sample cross-validation was conducted. The 393 respondents were randomly divided into an exploratory subsample (*n* = 196) and a holdout validation subsample (*n* = 197). Principal-axis EFA with Promax rotation was conducted in the exploratory subsample, whereas the prespecified CFA measurement model was estimated in the validation subsample. After this supplementary cross-validation, the final CFA and structural model were estimated using the full sample to maximize estimation precision.

The direct effects of H1a–H4b were estimated through the structural model. The indirect effects specified in H5a1–H5d2 were evaluated using 5,000 bootstrap resamples, with statistical significance determined from 95% bias-corrected bootstrap confidence intervals. Finally, common method bias was assessed using Harman’s single-factor test, a one-factor CFA comparison, a marker-variable partial-correlation check, and an additional CFA-based common latent method-factor analysis.

## 4 Results

### 4.1 Sample characteristics

The final analytic sample comprised 393 respondents who met the two screening conditions of experience with AI emotional companion apps and use at least twice per week. Sample characteristics are reported in Table 1.

**Table 1:** Sample characteristics (*N* = 393).

Category	Level	*n*	%
Gender	Male	154	39.2
	Female	239	60.8
Age	18–22	150	38.2
	23–27	160	40.7
	28–34	66	16.8
	35–39	14	3.6
	40 or older	3	0.8
Education	High school or below	29	7.4
	Junior college	75	19.1
	Four-year university	258	65.6
	Master’s	27	6.9
	Doctorate or above	4	1.0
Region	First-tier city	72	18.3
	New first-tier city	119	30.3
	Second-tier city	123	31.3
	Third-tier or below/rural	79	20.1
Relationship status	Unmarried/single	232	59.0
	In a relationship	114	29.0
	Married (incl. cohabiting)	33	8.4
	Divorced/separated	6	1.5
	No response	8	2.0
Length of use	Less than 1 month	50	12.7
	1–3 months	116	29.5
	3–6 months	111	28.2
	6 months–1 year	78	19.8
	More than 1 year	38	9.7
Weekly frequency	Several times a day	108	27.5
	Once a day	112	28.5
	Twice a week	103	26.2
	3–6 times a week	70	17.8
Paid-purchase experience	Yes	265	67.4
	No	128	32.6
Primary purpose of use	Emotional comfort/loneliness relief	193	49.1
	New experience/self-exploration	76	19.3
	Stress relief/escape from reality	58	14.8
	Entertainment/passing time	66	16.8

Including no-response and other categories, each grouping sums to *N* = 393.

### 4.2 Reliability and descriptive statistics

Before estimating the final measurement model, the reverse-coded items were reverse-scored and evaluated using multiple diagnostic criteria, including corrected item-total correlations, changes in internal consistency, standardized factor loadings, and model-level fit. As shown in Table 2, their corrected item-total correlations ranged from 0.301 to 0.613. Although several reverse-coded items showed acceptable item-total correlations, retaining each of them reduced the internal consistency of the corresponding scale or subscale, with Cronbach’s α increasing by 0.013 to 0.068 after deletion. In the alternative CFA retaining all reverse-coded items, their standardized loadings ranged from 0.319 to 0.625, with six of the eight items loading below 0.50. Thus, the decision to exclude the reverse-coded items was not based on a single cutoff or on Cronbach’s α alone, but on the overall pattern of item-level and model-level diagnostics, which was consistent with possible wording-related method effects. The reverse-coded items were excluded from the primary measurement model before structural-model estimation.

**Table 2:** Diagnostics for reverse-coded items.

Scale	Reverse-coded item	Corrected item-total correlation	*α* if retained	*α* after deletion	Δ*α*
ANT	ANT6R	0.371	0.854	0.881	0.027
ER	ER6R	0.354	0.848	0.869	0.021
ESC	ESC6R	0.397	0.800	0.816	0.016
PI	PI6R	0.430	0.849	0.865	0.016
EECI	EECI6R	0.613	0.929	0.943	0.013
CI	CI5R	0.301	0.802	0.870	0.068
SIG	SIG6R	0.396	0.869	0.899	0.030
LON	LON6R	0.497	0.918	0.942	0.024

Cronbach’s *α* was used to assess internal consistency. All constructs exceeded the recommended threshold of 0.70 (82), ranging from *α* = 0.816 to 0.945. The lower-order dimensions of the emotional bond with the AI—anthropomorphism (*α* = 0.881), emotional responsiveness (*α* = 0.869), and parasocial intimacy (*α* = 0.865)—all showed good internal consistency.

In the descriptive statistics, the means of the principal variables generally exceeded the scale midpoint. The emotional bond with the AI (*M* = 5.69) and continuance intention (*M* = 5.77), in particular, were relatively high, indicating that respondents reported relatively high levels of emotional bonding with AI emotional companion apps and relatively strong continuance intention. Loneliness (*M* = 5.00), actual-ideal self-discrepancy (*M* = 5.40), escapism (*M* = 5.49), and experience-enhancing consumption intention (*M* = 5.32) were all relatively high as well. Skewness and kurtosis fell within acceptable ranges, indicating that the distribution of the data was suitable for the subsequent multivariate analyses. Detailed results are presented in Table 3.

**Table 3:** Descriptive statistics and reliability of principal variables (*N* = 393).

Abbr.	Variable	Items	*M*	SD	Skew/Kurt	*α*
LON	Loneliness	5	4.999	1.488	−0.88/0.00	0.942
SIG	Actual-ideal self-discrepancy	5	5.400	1.147	−1.11/1.34	0.899
BOND	Emotional bond with the AI	15	5.685	0.849	−1.01/1.99	0.945
ESC	Escapism	5	5.485	1.011	−0.88/0.98	0.816
EECI	Experience-enhancing consumption intention	5	5.324	1.359	−1.20/1.25	0.943
CI	Continuance intention	4	5.769	0.923	−1.14/2.28	0.870

#### 4.2.1 Exploratory factor analysis

A common-factor exploratory factor analysis using principal axis factoring with Promax rotation was conducted to examine the latent dimensional structure of the 39 retained measurement items. Sampling adequacy was very good (KMO = 0.953), and Bartlett’s test of sphericity was significant (*χ*^2^ = 11,436.3, *p* < 0.001). Because the measurement framework specified six principal constructs, a six-factor solution was examined. All 39 items showed their largest loading on the theoretically corresponding factor, supporting the intended dimensional structure. Primary factor loadings ranged from 0.378 to 0.867. ESC5 showed a marginal primary loading (0.378), while ESC4 showed some secondary loading on the emotional-bond factor, consistent with the empirical overlap between escapism and emotional bonding observed in the subsequent discriminant-validity analysis. Overall, the common-factor solution was broadly consistent with the theoretically specified measurement structure.

A separate principal-axis factor analysis was also conducted on the 15 lower-order emotional-bond items (Table 4). The KMO was 0.952, and Bartlett’s test of sphericity was significant (*χ*^2^ = 3,716.3, *p* < 0.001). A one-factor solution was examined, and all 15 items loaded positively on the common factor, with factor loadings ranging from 0.662 to 0.767. The extracted factor accounted for approximately 53.5% of the total variance.

**Table 4:** Principal-axis factor analysis of the 15 emotional-bond items (*N* = 393).

Dimension	Item	Factor loading	Communality (*h*^2^)
Anthropomorphism	ANT1	0.767	0.589
	ANT2	0.745	0.554
	ANT3	0.748	0.559
	ANT4	0.729	0.531
	ANT5	0.751	0.564
Emotional responsiveness	ER1	0.756	0.571
	ER2	0.733	0.537
	ER3	0.752	0.565
	ER4	0.733	0.538
	ER5	0.702	0.493
Parasocial intimacy	PI1	0.754	0.569
	PI2	0.678	0.460
	PI3	0.662	0.438
	PI4	0.737	0.544
	PI5	0.714	0.509

Extraction method: principal axis factoring. A one-factor solution was examined for the 15 items representing the three lower-order dimensions of the emotional bond with the AI.

#### 4.2.2 Confirmatory factor analysis

The measurement model showed acceptable-to-good fit (*χ*^2^ = 1,241.86, df = 684, *χ*^2^/df = 1.82, CFI = 0.950, TLI = 0.946, RMSEA = 0.046, SRMR = 0.042). In particular, CFI was at 0.95, TLI was close to 0.95, and both RMSEA and SRMR were below the commonly cited 0.06 and 0.08 guidelines, respectively (83).

The hierarchical structure of the emotional bond with the AI was also supported. Anthropomorphism (*λ* = 0.928), emotional responsiveness (*λ* = 0.947), and parasocial intimacy (*λ* = 0.951) all loaded highly on the higher-order factor, indicating that the three lower-order dimensions converged on a single higher-order construct. This suggests that the emotional bond in AI emotional companion apps represents a multidimensional relational experience comprising anthropomorphism, emotional responsiveness, and parasocial intimacy. Most standardized item loadings ranged from 0.677 to 0.897; one escapism item (ESC5) had a marginally lower loading of 0.490. Because the escapism construct nevertheless showed acceptable composite reliability (CR = 0.837) and average variance extracted (AVE = 0.514), and because ESC5 directly captures the core notion of using the AI as “a means of escaping reality,” the item was retained to preserve the conceptual coverage of escapism. Across constructs, composite reliability ranged from 0.837 to 0.959 and average variance extracted from 0.514 to 0.887, supporting convergent validity. Detailed CFA results are presented in Table 5.

**Table 5:** Confirmatory factor analysis: standardized loadings, CR, and AVE (*N* = 393).

Variable	Item	*λ* (std.)	Cronbach’s *α*	CR	AVE
Loneliness	LON1	0.896	0.942	0.942	0.765
	LON2	0.881			
	LON3	0.892			
	LON4	0.863			
	LON5	0.840			
Actual-ideal self-discrepancy	SIG1	0.811	0.899	0.901	0.646
	SIG2	0.854			
	SIG3	0.790			
	SIG4	0.872			
	SIG5	0.677			
Anthropomorphism	ANT1	0.819	0.881	0.883	0.602
	ANT2	0.773			
	ANT3	0.748			
	ANT4	0.760			
	ANT5	0.777			
Emotional responsiveness	ER1	0.790	0.869	0.870	0.573
	ER2	0.758			
	ER3	0.758			
	ER4	0.749			
	ER5	0.727			
Parasocial intimacy	PI1	0.815	0.865	0.867	0.566
	PI2	0.714			
	PI3	0.705			
	PI4	0.774			
	PI5	0.747			
Escapism	ESC1	0.806	0.816	0.837	0.514
	ESC2	0.775			
	ESC3	0.749			
	ESC4	0.720			
	ESC5	0.490			
Experience-enhancing consumption intention	EECI1	0.897	0.943	0.943	0.767
	EECI2	0.856			
	EECI3	0.877			
	EECI4	0.884			
	EECI5	0.864			
Continuance intention	CI1	0.818	0.870	0.872	0.629
	CI2	0.794			
	CI3	0.757			
	CI4	0.803			
Emotional bond with the AI (BOND, 2nd order)	ANT/ER/PI	0.928/0.947/0.951	0.945	0.959	0.887

*λ* = standardized factor loading (all *p* < 0.001). CR, composite reliability; AVE, average variance extracted. In the BOND (2nd-order) row, the *λ* values are the second-order loadings of anthropomorphism, emotional responsiveness, and parasocial intimacy.

To examine the internal structure of the emotional bond with the AI, the second-order specification—three lower-order dimensions integrated under a higher-order factor—was compared with a model that merged anthropomorphism, emotional responsiveness, and parasocial intimacy into a single first-order factor (Table 6). The second-order specification fit marginally better (CFI = 0.950 vs. 0.939). Consistent with the exploratory analysis, in which the fifteen items converged on a single factor, we interpret this not as evidence that the three dimensions are sharply distinct, but as indicating that they are highly related facets most parsimoniously represented as lower-order components of one higher-order bond.

**Table 6:** Comparison of competing measurement models (*N* = 393).

Model	*χ* ^2^	df	*χ*^2^/df	CFI	TLI	RMSEA	SRMR
Proposed model (BOND second-order)	1,241.86	684	1.82	0.950	0.946	0.046	0.042
Six-factor (BOND merged into one first-order)	1,365.98	687	1.99	0.939	0.934	0.050	0.043
One-factor (all items on a single factor)	5,406.91	702	7.70	0.578	0.554	0.131	0.116
Reference value for acceptable fit	—	—	<3.00	≥0.90	≥0.90	≤0.08	≤0.08
Reference value for good fit	—	—	<2.00	≥0.95	≥0.95	≤0.06	≤0.06

A one-factor model loading all items onto a single factor showed very poor fit (CFI = 0.578, TLI = 0.554, RMSEA = 0.131, SRMR = 0.116) and was rejected, indicating that a single common factor did not adequately represent the covariance among all measurement items. This comparison therefore provides evidence against a single-factor representation rather than, by itself, establishing discriminant validity among the six principal constructs. Discriminant validity was assessed separately using the Fornell–Larcker criterion and HTMT ratios, as reported in Section 4.3 and Table 7.

**Table 7:** Correlations and discriminant validity (*N* = 393).

Variable	1	2	3	4	5	6
LON	(0.875)	0.561	0.364	0.469	0.244	0.391
SIG	0.52***	(0.804)	0.467	0.477	0.301	0.518
BOND	0.34***	0.43***	(0.942)	0.795	0.629	0.793
ESC	0.42***	0.41***	0.69***	(0.717)	0.473	0.618
EECI	0.23***	0.28***	0.59***	0.41***	(0.876)	0.602
CI	0.35***	0.46***	0.72***	0.51***	0.55***	(0.793)

Diagonal values in parentheses are the square roots of each construct’s AVE. The lower triangle reports Pearson correlations; the upper triangle reports HTMT (heterotrait–monotrait) ratios. All HTMT values are below the 0.85 threshold. ***p < 0.001.

#### 4.2.3 Robustness check including reverse-coded items

To examine whether the exclusion of the reverse-coded items materially affected the results, an alternative model was estimated in which all eight reverse-coded items were retained after reverse scoring. The results of the robustness comparison are presented in Supplementary Table S2. The 47-item measurement model showed *χ*^2^/df = 2.04, CFI = 0.914, TLI = 0.908, RMSEA = 0.051, and SRMR = 0.053, compared with *χ*^2^/df = 1.82, CFI = 0.950, TLI = 0.946, RMSEA = 0.046, and SRMR = 0.042 for the final 39-item model. Thus, retaining the reverse-coded items resulted in a noticeable deterioration in incremental fit, although the overall model remained interpretable. Both SRMR values remained below the conventional 0.08 guideline, indicating that the residual fit of the 47-item model was still acceptable even though its incremental fit was weaker. More importantly, the substantive structural pattern was highly stable across the two specifications. With the reverse-coded items retained, the standardized coefficients were 0.188 for LON → BOND, 0.374 for SIG → BOND, 0.313 for LON → ESC, 0.284 for SIG → ESC, 0.660 for BOND → EECI, −0.021 for ESC → EECI, 0.806 for BOND → CI, and 0.021 for ESC → CI. These estimates closely paralleled those obtained from the 39-item model (0.177, 0.391, 0.293, 0.305, 0.667, −0.020, 0.807, and 0.027, respectively). Accordingly, excluding the reverse-coded items improved measurement quality and model fit but did not materially alter the central competing-pathway pattern.

#### 4.2.4 Split-sample cross-validation

To address the possibility that conducting exploratory and confirmatory measurement analyses on the same sample could capitalize on sample-specific characteristics, an additional split-sample cross-validation was conducted. The full sample was randomly divided into an exploratory subsample (*n* = 196) and an independent validation subsample (*n* = 197). In the exploratory subsample, principal-axis factor analysis with Promax rotation showed adequate sampling suitability (KMO = 0.934; Bartlett’s *χ*^2^ = 5,931.50, *p* < 0.001). All 39 retained items had their largest loading on the theoretically corresponding factor, with primary loadings ranging from 0.392 to 0.866. The prespecified second-order CFA model was then estimated independently in the validation subsample. The model showed acceptable fit (*χ*^2^ = 1,108.29, df = 684, *χ*^2^/df = 1.62, CFI = 0.926, TLI = 0.920, RMSEA = 0.056, SRMR = 0.057). Standardized item loadings ranged from 0.531 to 0.906, and the second-order loadings of anthropomorphism, emotional responsiveness, and parasocial intimacy on the emotional bond with the AI were 0.912, 0.938, and 0.931, respectively. These results indicate that the principal measurement structure was reproduced in the holdout validation subsample rather than being specific to the full-sample analysis. The full sample was subsequently retained for the final CFA and structural-model estimates to maximize statistical precision. Detailed results of the split-sample cross-validation are reported in Supplementary Table S3.

### 4.3 Correlations and discriminant validity

In the Pearson correlation analysis (Table 7), all correlations among the principal variables were statistically significant (*p* < 0.001), and their overall direction matched the hypotheses. Loneliness and actual-ideal self-discrepancy both correlated positively with the emotional bond with the AI (*r* = 0.34 and *r* = 0.43) and with escapism (*r* = 0.42 and *r* = 0.41). This suggests that users reporting higher levels of loneliness or actual-ideal self-discrepancy may display both an emotional bond with the AI and an avoidant use tendency. The emotional bond with the AI showed strong positive correlations with experience-enhancing consumption intention (*r* = 0.59) and continuance intention (*r* = 0.72), and escapism also correlated significantly and positively with the two outcome variables (*r* = 0.41 and *r* = 0.51). The correlations were generally larger for the emotional bond with the AI, however, suggesting that the relational route may be more closely tied to consumer responses than the avoidant route.

In the discriminant validity assessment, the square root of each construct’s AVE exceeded its correlations under the Fornell–Larcker criterion (84), and the heterotrait-monotrait ratio (HTMT; 87) ranged from 0.244 to 0.795, all below the 0.85 threshold (upper triangle of Table 7). These results support the empirical distinctness of the principal constructs and the discriminant validity of the measurement model. At the same time, the association between the emotional bond with the AI and escapism was relatively high (*r* = 0.69; HTMT = 0.795). Although this value remained below the 0.85 criterion and therefore did not indicate a formal violation of discriminant validity, it suggests that the two proposed routes are empirically intertwined rather than cleanly orthogonal. This overlap is theoretically plausible in the AI-companion context, because users may experience escape from reality partly through a relationship-like bond with an emotionally responsive AI. Therefore, the two constructs are treated as theoretically distinguishable but empirically closely related pathways.

### 4.4 Structural model and direct effects

As reported in Table 8, the structural model showed acceptable fit on several conventional indices (*χ*^2^ = 1,464.68, df = 690, *χ*^2^/df = 2.12, CFI = 0.930, TLI = 0.925, RMSEA = 0.054), although the SRMR (0.109) exceeded the commonly used 0.08 guideline, indicating some residual misfit. The CFI and TLI exceeded the conventional 0.90 threshold but did not reach the more stringent 0.95 criterion. Accordingly, the overall fit should be regarded as acceptable on several indices but not uniformly strong, and the structural results should be interpreted with appropriate caution. The elevated SRMR may partly reflect the constrained structural specification, in which the two mediators were modeled as parallel routes despite their substantial empirical overlap (*r* = 0.693), leaving some covariance between the emotional-bond and escapism indicators unexplained by the directional paths. Consistent with this interpretation, the corresponding measurement model, which freely estimates the correlation between the two constructs, yielded an SRMR of 0.042.

**Table 8:** Structural model direct effects (standardized path coefficients, *N* = 393).

Path	*B*	*β*	SE	C.R.	*p*
Emotional bond with the AI (*R*^2^ = 0.261) ← Loneliness	0.102	0.177**	0.035	2.95	0.003
← Actual-ideal self-discrepancy	0.298	0.391***	0.049	6.11	<0.001
Escapism (*R*^2^ = 0.278) ← Loneliness	0.191	0.293***	0.041	4.67	<0.001
← Actual-ideal self-discrepancy	0.262	0.305***	0.055	4.74	<0.001
Experience-enhancing consumption intention (*R*^2^ = 0.438) ← Emotional bond with the AI	1.045	0.667***	0.086	12.20	<0.001
← Escapism	−0.028	−0.020	0.064	−0.43	0.665
Continuance intention (*R*^2^ = 0.664) ← Emotional bond with the AI	0.810	0.807***	0.059	13.62	<0.001
← Escapism	0.024	0.027	0.037	0.65	0.515

*B*, unstandardized coefficient; *β*, standardized coefficient. **p < 0.01, ***p < 0.001. Model fit: *χ*^2^/df = 2.12, CFI = 0.930, TLI = 0.925, RMSEA = 0.054. SRMR = 0.109.

Regarding the associations between psychological vulnerability and the mediators, loneliness (*β* = 0.177, *p* = 0.003) and actual-ideal self-discrepancy (*β* = 0.391, *p* < 0.001) were both positively and significantly associated with the emotional bond with the AI (*R*^2^ = 0.261), and loneliness (*β* = 0.293, *p* < 0.001) and actual-ideal self-discrepancy (*β* = 0.305, *p* < 0.001) were also positively and significantly associated with escapism (*R*^2^ = 0.278). H1a, H1b, H2a, and H2b were thus supported.

Regarding the associations with consumer responses, the emotional bond with the AI showed strong positive associations with both experience-enhancing consumption intention (*β* = 0.667, *p* < 0.001) and continuance intention (*β* = 0.807, *p* < 0.001). The model explained 43.8% of the variance in EECI and 66.4% of the variance in CI. H3a and H3b were therefore supported. These results suggest that, although psychological vulnerability was positively associated with both the relational and avoidant compensation routes, the route more strongly associated with consumer responses appears to be the emotional bond with the AI rather than escapism.

Because the emotional bond with the AI and escapism were strongly correlated, additional collinearity diagnostics were conducted to examine whether the nonsignificant effects of escapism were attributable to severe multicollinearity. Variance inflation factors (VIFs) were calculated using construct-score regressions corresponding to the predictor sets in the structural equations.

As shown in Table 9, the VIFs for loneliness and actual-ideal self-discrepancy were 1.363 in the equations predicting the emotional bond with the AI and escapism. The VIFs for the emotional bond with the AI and escapism were 1.926 in the equations predicting experience-enhancing consumption intention and continuance intention. These values were below conventional cutoffs, indicating that the structural estimates were not seriously distorted by severe multicollinearity. Nevertheless, the relatively high association between the emotional bond with the AI and escapism indicates substantial shared variance. Therefore, the nonsignificant escapism effects should be interpreted as limited unique explanatory power after accounting for the emotional bond, rather than as evidence that escapism is irrelevant to AI-companion use. As an additional diagnostic, when LON, SIG, BOND, and ESC were entered together, the VIFs ranged from 1.465 to 2.077, further indicating that severe multicollinearity was not present.

**Table 9:** Collinearity diagnostics for structural predictor sets.

Endogenous variable	Predictor set	Predictor	Tolerance	VIF
BOND	LON, SIG	LON	0.734	1.363
BOND	LON, SIG	SIG	0.734	1.363
ESC	LON, SIG	LON	0.734	1.363
ESC	LON, SIG	SIG	0.734	1.363
EECI	BOND, ESC	BOND	0.519	1.926
EECI	BOND, ESC	ESC	0.519	1.926
CI	BOND, ESC	BOND	0.519	1.926
CI	BOND, ESC	ESC	0.519	1.926

### 4.5 Mediation analysis

Mediation was tested through the indirect effects among latent variables within the structural model, with significance judged by 95% bias-corrected confidence intervals based on 5,000 bootstrap resamples (Table 10). All four indirect paths through the emotional bond with the AI were significant. Loneliness had significant indirect effects, via the emotional bond with the AI, on experience-enhancing consumption intention (indirect effect = 0.118, 95% CI [0.015, 0.225]) and continuance intention (indirect effect = 0.143, 95% CI [0.016, 0.268]); actual-ideal self-discrepancy likewise had significant indirect effects, via the emotional bond with the AI, on experience-enhancing consumption intention (indirect effect = 0.261, 95% CI [0.138, 0.428]) and continuance intention (indirect effect = 0.315, 95% CI [0.168, 0.503]). H5a1, H5a2, H5b1, and H5b2 were therefore supported.

**Table 10:** Mediation results (structural-model indirect effects, 5,000 bootstrap resamples, *N* = 393).

Independent	Mediator	Dependent	Indirect	Boot LLCI	Boot ULCI	Result
Loneliness	Emotional bond	EECI	0.118	0.015	0.225	Supported
Loneliness	Emotional bond	CI	0.143	0.016	0.268	Supported
Actual-ideal self-discrepancy	Emotional bond	EECI	0.261	0.138	0.428	Supported
Actual-ideal self-discrepancy	Emotional bond	CI	0.315	0.168	0.503	Supported (partial mediation)
Loneliness	Escapism	EECI	−0.006	−0.043	0.047	Rejected
Loneliness	Escapism	CI	0.008	−0.035	0.073	Rejected
Actual-ideal self-discrepancy	Escapism	EECI	−0.006	−0.052	0.042	Rejected
Actual-ideal self-discrepancy	Escapism	CI	0.008	−0.033	0.086	Rejected

Indirect effects are standardized estimates computed as the product of structural path coefficients (a × b). The 95% confidence intervals (LLCI–ULCI) are bias-corrected intervals from 5,000 bootstrap resamples; an interval excluding zero indicates significance. EECI, experience-enhancing consumption intention; CI, continuance intention.

By contrast, all four indirect paths through escapism were nonsignificant. That is, although psychological vulnerability was positively associated with escapism, the escapism route was not indirectly associated with experience-enhancing consumption intention or continuance intention as an independent mediating pathway. H5c1, H5c2, H5d1, and H5d2 were therefore rejected. H5b2 was supported, but the relationship was partially rather than fully mediated because the residual direct effect from actual-ideal self-discrepancy to continuance intention remained significant. To further distinguish between full and partial mediation, these indirect effects were considered together with the partial-mediation model reported in Section 4.5.2. When direct paths from loneliness and actual-ideal self-discrepancy to both outcomes were added, the direct effects of loneliness on experience-enhancing consumption intention and continuance intention, as well as the direct effect of actual-ideal self-discrepancy on experience-enhancing consumption intention, were nonsignificant. These relationships were therefore consistent with full mediation through the specified mediators. In contrast, the direct association between actual-ideal self-discrepancy and continuance intention remained significant (*β* = 0.144, *p* = 0.007), while its indirect effect through the emotional bond with the AI also remained significant. This pattern indicates partial rather than full mediation for the relationship between actual-ideal self-discrepancy and continuance intention.

In sum, among the two competing mediating routes, only the emotional bond with the AI showed significant indirect associations with experience-enhancing consumption intention and continuance intention. However, the significant residual direct association between actual-ideal self-discrepancy and continuance intention indicates that the emotional-bond pathway does not fully account for this particular relationship. Thus, the relational compensation route represents the primary indirect pathway, but it is not exhaustive for explaining the link between actual-ideal self-discrepancy and continuance intention.

#### 4.5.1 Common method bias

Because all substantive variables were measured through self-report from the same respondents at a single time point, the possibility of common method bias was examined using multiple complementary diagnostics (85). Harman’s single-factor test showed that the first unrotated factor accounted for 40.18% of the total variance. Because Harman’s test provides only a limited diagnostic and cannot establish the absence of common method variance (85), additional assessments were conducted.

First, the proposed measurement structure was compared with a one-factor CFA model. The one-factor model showed very poor fit (CFI = 0.578, RMSEA = 0.131; Table 6), indicating that a single factor did not adequately account for the covariance among the substantive items. Second, a marker-variable partial-correlation check was conducted. The marker variable consisted of three items assessing general decision-making prudence and was selected because its content was conceptually outside the focal domain of AI-companion use and the substantive constructs examined in this study. However, its correlations with the focal constructs ranged from *r* = 0.169 to 0.460. Thus, although the marker was theoretically distal, it was not empirically orthogonal to the substantive variables. The marker-based analysis was therefore treated only as a supplementary sensitivity check rather than as a definitive statistical correction for common method variance. As shown in Table 11, the principal zero-order relationships remained significant after controlling for the marker variable.

**Table 11:** Marker-variable partial-correlation check.

Relationship	Zero-order *r*	Partial *r* controlling MARK
LON-BOND	0.343***	0.304***
SIG-BOND	0.430***	0.338***
LON-ESC	0.417***	0.386***
SIG-ESC	0.408***	0.323***
BOND-EECI	0.594***	0.538***
BOND-CI	0.720***	0.649***
ESC-EECI	0.411***	0.333***
ESC-CI	0.514***	0.408***
BOND-ESC	0.693***	0.630***

MARK, marker variable measuring general decision-making prudence. Partial correlations controlled for MARK. ***p < 0.001.

To provide a stronger CFA-based assessment, an additional common latent method-factor model was estimated. In this model, each of the 39 substantive indicators loaded on both its theoretically specified construct and an orthogonal common method factor. The correlated-trait CFA without the method factor showed acceptable fit (*χ*^2^ = 1,200.51, df = 674, CFI = 0.953, TLI = 0.948, RMSEA = 0.045), whereas the model including the common method factor showed improved fit (*χ*^2^ = 1,044.93, df = 635, CFI = 0.963, TLI = 0.957, RMSEA = 0.041; Δ*χ*^2^(39) = 155.58, *p* < 0.001). This improvement indicates the presence of statistically detectable common method variance. However, the squared standardized method loadings accounted for an average of approximately 2.9% of item variance (median = 2.2%), while the substantive standardized loadings remained between 0.480 and 0.900 and changed by only 0.019 on average in absolute terms. Thus, the common method component was detectable but did not appear to dominate the substantive measurement structure. Accordingly, common method bias cannot be fully ruled out, and the results should be interpreted with appropriate caution. Detailed model-fit results for the CFA-based common latent method-factor assessment are reported in Supplementary Table S4.

#### 4.5.2 Robustness checks: competing-mediation contrasts, partial mediation, and the escapism-only model

Because the central claim concerns the relative strength of the two routes, the difference between each pair of indirect effects (relational minus avoidant) was tested directly with bias-corrected 95% confidence intervals from 5,000 bootstrap resamples (Table 12). All four contrasts excluded zero, indicating that the indirect effect through the emotional bond was significantly larger than that through escapism for both antecedents and both outcomes. This provides a formal test of the asymmetry between the routes, beyond the observation that one was significant and the other was not.

**Table 12:** Competing-mediation contrast tests (relational − avoidant indirect effects, standardized, 5,000 bias-corrected bootstrap resamples, *N* = 393).

Contrast (relational − avoidant)	*Δ*	95% CI (BC)	Result
LON → BOND → EECI vs. LON → ESC → EECI	0.124	[0.016, 0.269]	Relational > Avoidant
LON → BOND → CI vs. LON → ESC → CI	0.135	[0.005, 0.301]	Relational > Avoidant
SIG → BOND → EECI vs. SIG → ESC → EECI	0.267	[0.124, 0.465]	Relational > Avoidant
SIG → BOND → CI vs. SIG → ESC → CI	0.307	[0.148, 0.512]	Relational > Avoidant

Δ = difference between the relational indirect effect (through the emotional bond with the AI) and the avoidant indirect effect (through escapism). Bias-corrected 95% confidence intervals excluding zero indicate that the relational indirect effect is significantly larger. BOND = emotional bond with the AI; ESC, escapism; EECI, experience-enhancing consumption intention; CI, continuance intention.

To assess whether the associations between the antecedents and the outcomes were fully accounted for by the specified mediating pathways, a partial-mediation model adding direct paths from loneliness and actual-ideal self-discrepancy to both outcomes was estimated. Its fit was virtually identical to the full-mediation model (CFI = 0.931, TLI = 0.926, RMSEA = 0.053), and the bond and escapism paths were essentially unchanged. Three of the four added direct paths were nonsignificant (loneliness → experience-enhancing consumption intention, *β* = 0.012; actual-ideal self-discrepancy → experience-enhancing consumption intention, *β* = −0.012; loneliness → continuance intention, *β* = 0.043; all n.s.), consistent with full mediation. The exception was a significant direct path from actual-ideal self-discrepancy to continuance intention (*β* = 0.144, *p* = 0.007), indicating partial rather than full mediation for that single path.

Finally, to probe why the escapism route was nonsignificant despite escapism’s substantial zero-order correlations with the outcomes, a model with escapism as the sole mediator was estimated. With the emotional bond removed, escapism was significantly associated with both experience-enhancing consumption intention (*β* = 0.530, *p* < 0.001) and continuance intention (*β* = 0.670, *p* < 0.001); these associations became nonsignificant once the emotional bond was reintroduced. Together with the relatively high bond–escapism association (*r* = 0.69; HTMT = 0.795), this pattern indicates that the null escapism result should not be interpreted as evidence that escapism is irrelevant to AI-companion use. Rather, escapism appears to share substantial variance with the emotional bond, and its association with consumer responses is largely absorbed by the relational bond when both routes are modeled simultaneously. Thus, the result is best understood as indicating that escapism has limited unique explanatory power beyond the emotional bond, not that avoidant immersion has no role in the user experience.

#### 4.5.3 Supplementary analysis by paid-purchase experience

To address whether the consumption-intention results differed by actual paid-purchase experience, a supplementary construct-score analysis was conducted by comparing respondents with prior paid-purchase experience (*n* = 265) and non-payers (*n* = 128). Independent-samples tests showed that paid users reported significantly higher scores than non-payers on loneliness, actual-ideal self-discrepancy, emotional bond with the AI, escapism, experience-enhancing consumption intention, and continuance intention. As shown in Table 13, the largest difference was observed for experience-enhancing consumption intention (paid users: *M* = 5.78, SD = 0.91; non-payers: *M* = 4.37, SD = 1.62; Welch’s t = 9.16, *p* < 0.001, Cohen’s *d* = 1.19), indicating that the willingness-to-pay measure is meaningfully associated with respondents’ prior payment experience.

**Table 13:** Mean differences by paid-purchase experience.

Variable	Paid users *M*	Paid users SD	Non-payers *M*	Non-payers SD	*t*	df	*p*	Cohen’s *d*
LON	5.192	1.395	4.600	1.597	3.584	223.36	<0.001	0.404
SIG	5.482	1.223	5.230	0.955	2.236	313.37	0.026	0.221
BOND	5.835	0.771	5.375	0.920	4.886	215.92	<0.001	0.559
ESC	5.587	0.978	5.275	1.050	2.824	235.97	0.005	0.312
EECI	5.783	0.908	4.373	1.623	9.162	166.51	<0.001	1.187
CI	5.919	0.850	5.459	0.993	4.503	219.58	<0.001	0.512

We further examined whether the key paths differed between paid users and non-payers using supplementary construct-score regression and interaction tests (Table 14). In both groups, the emotional bond with the AI was positively associated with experience-enhancing consumption intention, whereas escapism was not significantly associated with this outcome. The interaction terms for paid-purchase experience were nonsignificant for both BOND × PAY → EECI (B = 0.226, *p* = 0.187) and ESC × PAY → EECI (B = 0.024, *p* = 0.871), suggesting that the key consumption-intention paths did not significantly differ between paid users and non-payers. For continuance intention, however, the interaction term was significant (ESC × PAY → CI, B = 0.260, *p* = 0.006): escapism was weakly but positively associated with continuance intention among paid users (*β* = 0.133, *p* = 0.024) and nonsignificant among non-payers (*β* = −0.152, *p* = 0.092). Even in the paid-user group, the coefficient for the emotional bond with the AI (*β* = 0.619) was substantially larger than that for escapism, so this difference qualifies the magnitude rather than the direction of the competing-pathway pattern. These results support the robustness of the relational pathway while confirming that the outcome should be interpreted as experience-enhancing consumption intention rather than actual expenditure.

**Table 14:** Supplementary path analysis by paid-purchase experience.

Path	Paid users *β*	Paid *p*	Non-payers *β*	Non-payers *p*	Interaction *B*	Interaction *p*	Group diff
LON → BOND	0.153*	0.020	0.100	0.280	0.027	0.657	ns
SIG → BOND	0.367***	<0.001	0.333***	<0.001	−0.089	0.319	ns
LON → ESC	0.255***	<0.001	0.289**	0.002	−0.011	0.876	ns
SIG → ESC	0.307***	<0.001	0.170	0.067	0.059	0.585	ns
BOND→EECI	0.747***	<0.001	0.370**	0.002	0.226	0.187	ns
ESC → EECI	0.032	0.550	0.004	0.972	0.024	0.871	ns
BOND → CI	0.619***	<0.001	0.801***	< 0.001	−0.183	0.098	ns
ESC → CI	0.133*	0.024	−0.152	0.092	0.260	0.006	Sig.

β, standardized coefficient; B, unstandardized interaction coefficient. *p < 0.05, **p < 0.01, ***p < 0.001; ns, nonsignificant; Sig, significant.

#### 4.5.4 Sensitivity analyses by demographic subgroups

To examine whether the competing-pathway pattern was driven by a specific demographic subgroup, supplementary sensitivity analyses were conducted by gender, age group, and relationship status. Because the sample was concentrated among young adults, age was recoded into two groups: 18–27 years and 28 years or older. Relationship status was recoded as single/unmarried versus non-single, and respondents who selected “no response” were excluded from this subgroup analysis.

As shown in Table 15, the emotional bond with the AI remained positively and significantly associated with both experience-enhancing consumption intention and continuance intention across all gender, age, and relationship-status subgroups. Escapism was generally nonsignificant once the emotional bond was included, with one exception: among respondents aged 28 years or older, escapism was also positively associated with experience-enhancing consumption intention. Even in this subgroup, however, the standardized coefficient for the emotional bond was larger than that for escapism. These results suggest that the dominance of the relational pathway was not driven by a single demographic subgroup, although the older subgroup indicates that escapism may retain some consumption-related relevance for certain users. Because several subgroup sizes were modest, these analyses should be interpreted as sensitivity checks rather than formal multi-group invariance tests. Additional interaction tests showed that the key relational paths were not significantly moderated by gender, age group, or relationship status. The only significant interaction concerned the escapism path to experience-enhancing consumption intention by relationship status, but this did not alter the overall pattern that the emotional bond showed the stronger association with the outcomes across subgroups.

**Table 15:** Sensitivity analyses across demographic subgroups.

Grouping variable	Subgroup	*N*	BOND → EECI	ESC → EECI	BOND → CI	ESC → CI
Gender	Male	154	0.618***	−0.096	0.749***	0.039
Gender	Female	239	0.578***	0.047	0.678***	0.020
Age	18–27	310	0.601***	−0.044	0.732***	0.026
Age	28+	83	0.402**	0.291*	0.507**	0.057
Relationship status	Single/unmarried	232	0.664***	−0.116	0.762***	−0.025
Relationship status	Non-single	153	0.505***	0.153	0.633***	0.087

Values are standardized coefficients (*β*). *p < 0.05, **p < 0.01, ***p < 0.001.

### 4.6 Summary of hypothesis testing

Overall, 10 of the 16 hypotheses were supported and 6 were rejected (Table 16). Loneliness and actual-ideal self-discrepancy were both positively associated with the emotional bond with the AI and escapism, supporting H1a, H1b, H2a, and H2b. This indicates that psychological vulnerability was associated with both relational and avoidant compensatory experiences.

**Table 16:** Summary of hypothesis testing.

Hypothesis	Content	Result
H1a	Loneliness will be positively associated with the emotional bond with the AI.	Supported
H1b	Actual-ideal self-discrepancy will be positively associated with the emotional bond with the AI.	Supported
H2a	Loneliness will be positively associated with escapism.	Supported
H2b	Actual-ideal self-discrepancy will be positively associated with escapism.	Supported
H3a	The emotional bond with the AI will be positively associated with experience-enhancing consumption intention.	Supported
H3b	The emotional bond with the AI will be positively associated with continuance intention.	Supported
H4a	Escapism will be positively associated with experience-enhancing consumption intention.	Rejected
H4b	Escapism will be positively associated with continuance intention.	Rejected
H5a1	The emotional bond with the AI will statistically mediate the association between loneliness and experience-enhancing consumption intention.	Supported
H5a2	The emotional bond with the AI will statistically mediate the association between loneliness and continuance intention.	Supported
H5b1	The emotional bond with the AI will statistically mediate the association between actual-ideal self-discrepancy and experience-enhancing consumption intention.	Supported
H5b2	The emotional bond with the AI will statistically mediate the association between actual-ideal self-discrepancy and continuance intention.	Supported (partial mediation)
H5c1	Escapism will statistically mediate the association between loneliness and experience-enhancing consumption intention.	Rejected
H5c2	Escapism will statistically mediate the association between loneliness and continuance intention.	Rejected
H5d1	Escapism will statistically mediate the association between actual-ideal self-discrepancy and experience-enhancing consumption intention.	Rejected
H5d2	Escapism will statistically mediate the association between actual-ideal self-discrepancy and continuance intention.	Rejected

Ten of the 16 hypotheses were supported and six were rejected. H5b2 was supported with partial rather than full mediation because the direct association between actual-ideal self-discrepancy and continuance intention remained significant.

The two mediators showed different relationships with the outcome variables. The emotional bond with the AI was significantly associated with both experience-enhancing consumption intention and continuance intention, supporting H3a and H3b. In contrast, escapism was not significantly associated with either outcome after the emotional bond was included, so H4a and H4b were rejected. The mediation results followed the same pattern: the indirect pathways through the emotional bond with the AI were significant, supporting H5a1, H5a2, H5b1, and H5b2, whereas the indirect pathways through escapism were nonsignificant, rejecting H5c1, H5c2, H5d1, and H5d2.

Taken together, the findings suggest that although psychological vulnerability was associated with both emotional bonding and escapism, only the relational compensation route showed significant indirect associations with experience-enhancing consumption intention and continuance intention. The avoidant route added little unique explanatory value once the emotional bond with the AI was modeled simultaneously.

## 5 Discussion

This study examined the associations of loneliness and actual-ideal self-discrepancy with experience-enhancing consumption intention and continuance intention through emotional bonding with the AI and escapism. Based on data from 393 active users, both loneliness and actual-ideal self-discrepancy were positively associated with emotional bonding and escapism. However, only the emotional-bond pathway showed significant indirect associations with the two outcomes, whereas escapism contributed little unique explanatory power once emotional bonding was considered simultaneously. These findings indicate that relational compensation is more closely associated with experience-enhancing consumption intention and continuance intention than avoidant compensation.

### 5.1 Interpretation of findings

The findings show that loneliness and actual-ideal self-discrepancy were positively associated with both emotional bonding with the AI and escapism, indicating that users experiencing relational or self-related deficits may engage with AI companions both as relational partners and as sources of temporary relief. The stronger association between actual-ideal self-discrepancy and emotional bonding is also consistent with the possibility that users who perceive a gap between their actual and ideal selves may be especially inclined toward self-related projection and compensatory engagement in virtual environments (21–25).

The two compensatory routes, however, differed sharply in their associations with consumer responses. Emotional bonding was strongly associated with both experience-enhancing consumption intention (*β* = 0.667) and continuance intention (*β* = 0.807), suggesting that willingness to pay for enriched experiences and continuance intention are closely associated with the relationship-like bond with the AI. This pattern is consistent with parasocial interaction research showing that emotionally meaningful relationships with mediated targets can support attachment, loyalty, and sustained engagement (11, 34).

Escapism, by contrast, was not significantly associated with either outcome once emotional bonding was included in the same model. Importantly, this does not imply that escapism is irrelevant. When modeled alone, escapism was significantly associated with both outcomes, but these associations became nonsignificant after emotional bonding was introduced. Together with the substantial association between the two mediators, this suggests that escapism shares considerable variance with emotional bonding and contributes limited unique explanatory power beyond the relational pathway. The mediation and contrast analyses further supported this asymmetry: all indirect effects through emotional bonding were significant, all indirect effects through escapism were nonsignificant, and the relational indirect effects were significantly stronger across all antecedent–outcome pairs.

One important qualification concerns actual-ideal self-discrepancy and continuance intention. In the partial-mediation model, actual-ideal self-discrepancy retained a significant direct association with continuance intention (*β* = 0.144, *p* = 0.007), indicating that emotional bonding does not fully account for this relationship. Overall, the findings suggest that AI-companion consumption-related and continuance intentions are more closely associated with relational compensation than with avoidant compensation, while additional mechanisms may still connect self-discrepancy with continuance intention.

### 5.2 Theoretical implications

The study’s theoretical implications are as follows. First, this study positions loneliness and actual-ideal self-discrepancy as theoretically relevant psychological correlates of AI-companion consumption-related and continuance intentions. By integrating compensation theory, self-discrepancy theory, and uses and gratifications theory within a consumer-behavior framework, it shows how these self-reported vulnerabilities are associated with relational and consumption-related responses. This extends the study of AI companions from questions of use and psychological effects toward a more specific account of how loneliness and self-discrepancy are linked to willingness to pay for enriched experiences and continuance intention.

Second, this study refines the compensatory-use perspective by distinguishing relational compensation from avoidant compensation as two theoretically different pathways. Rather than treating compensatory media use as a unitary response to psychological deficits, the model shows that compensation can involve either forming a relationship-like bond with an AI or seeking temporary distance from real-world discomfort. This distinction extends compensation theory by suggesting that the form of compensation matters for how psychological vulnerability is linked to downstream consumer responses. In the AI-companion context, relational and avoidant compensation are therefore better understood as related but non-equivalent mechanisms, providing a more differentiated account of how users respond to unmet relational or self-related needs.

Third, this study advances the conceptualization of emotional bonding with AI by modeling anthropomorphism, emotional responsiveness, and parasocial intimacy as lower-order dimensions of a higher-order relational construct. This integrative structure provides a more comprehensive representation of how users experience relationship-like connection with AI companions and offers a parsimonious framework for examining its role in experience-enhancing consumption intention and continuance intention.

Fourth, this study extends parasocial interaction theory into the consumer-behavior context of AI companionship by linking relationship-like bonding with consumption intention and continuance intention. It further shows that such relational engagement is associated with loneliness and actual-ideal self-discrepancy, connecting parasocial bonding not only to media attachment but also to psychologically grounded patterns of AI-companion consumption.

From a public mental health perspective, the contribution of the present study is indirect but relevant. The study does not demonstrate that AI-companion use improves or worsens mental health, nor does it identify a clinically vulnerable population. Instead, it shows that higher self-reported loneliness and actual-ideal self-discrepancy are associated with stronger emotional bonding with an AI companion, and stronger emotional bonding is also associated with greater willingness to pay for experience enhancement and stronger continuance intention. This pattern identifies a behavioral pathway through which higher levels of self-reported loneliness and actual-ideal self-discrepancy may become linked to deeper and more persistent engagement with emotionally responsive AI. Accordingly, the public mental health relevance lies in informing prevention-oriented user protection and responsible relational design, rather than in establishing direct mental health effects.

### 5.3 Practical implications

First, relational bonding showed a stronger unique association with consumer responses than escapism. In the simultaneous model, emotional bonding was strongly associated with experience-enhancing consumption intention and continuance intention, whereas escapism showed limited unique explanatory power after accounting for emotional bonding. This suggests that relationship-oriented design features—such as cues supporting humanlikeness, emotional responsiveness, and intimacy—may be more relevant to the consumer responses examined here than features emphasizing momentary escape or stimulation. From a managerial perspective, these findings suggest that platform operators may benefit from paying greater attention to the quality and durability of relational experiences, rather than relying primarily on momentary stimulation or escapist engagement. Accordingly, anthropomorphism, emotional responsiveness, and parasocial intimacy may represent relevant design considerations for platforms seeking to support continuance intention and experience-enhancing consumption intention.

Second, the findings highlight the importance of considering users who report higher levels of loneliness or actual-ideal self-discrepancy. Users reporting higher levels of loneliness and actual-ideal self-discrepancy also reported stronger emotional bonding with the AI, and this stronger bonding was associated with higher experience-enhancing consumption intention and continuance intention. In particular, the stronger association between actual-ideal self-discrepancy and emotional bonding suggests that users reporting greater self-discrepancy, alongside those reporting greater loneliness, may warrant particular attention in future user-segmentation research and responsible platform design. These descriptive patterns—the prominence of emotional comfort/loneliness relief as a primary purpose of use and the high prevalence of prior paid-purchase experience—further suggest the practical relevance of considering heterogeneity in users’ psychological characteristics.

Third, these findings have implications for preventive user protection. Users reporting higher levels of loneliness or actual-ideal self-discrepancy showed stronger emotional bonding with the AI, and stronger emotional bonding was also associated with greater experience-enhancing consumption intention and continuance intention. From a corporate standpoint, this may be read as an important market opportunity, but it also carries the implication that vigilance is needed regarding the possibility that psychological deficits such as loneliness and actual-ideal self-discrepancy could be commercially exploited. Recent critical research has raised concerns that AI companion products may foster emotional dependence and commodify intimacy through affective attachments and engagement loops, particularly among users experiencing loneliness or relational vulnerability (86). Therefore, platform governance should not focus only on content safety or privacy protection, but should also address how intimacy-oriented design and payment architectures may create risks of heightened dependency among users who may already be experiencing greater loneliness or self-related distress. Platforms could therefore consider features for autonomous management of use time and spending, clear payment guidance, warnings about excessive use, and transparent disclosure, so that relationship-forming design does not heighten the risk of excessive immersion or overspending among users reporting higher levels of loneliness or self-discrepancy.

Fourth, escapism showed limited unique explanatory power for experience-enhancing consumption intention and continuance intention after accounting for emotional bonding. This suggests that managerial implications should remain focused on the consumer responses directly examined in this study, rather than on unmeasured behaviors such as session duration or actual retention.

### 5.4 Limitations and future research

Several limitations should be considered. First, the cross-sectional design does not establish temporal or causal ordering among loneliness, actual-ideal self-discrepancy, emotional bonding, escapism, and consumer responses. Reciprocal relationships remain possible, and future longitudinal or experimental research is needed to examine directionality more directly. Accordingly, the indirect effects reported in this study should be interpreted as model-based statistical indirect associations rather than evidence of causal mediation.

Second, escapism was modeled as a single-dimensional construct, although prior research distinguishes self-suppression and self-expansion forms of escapism (39). This may partly explain its empirical overlap with emotional bonding. Future studies should distinguish these dimensions to clarify whether avoidant escape and relational immersion operate differently in AI-companion use.

Third, the study relied primarily on self-report measures collected at a single time point. Although the latent method-factor analysis suggested that common method variance was limited, it was statistically detectable, and the marker variable was not empirically orthogonal to the focal constructs. In addition, although the substantive results were stable when the reverse-coded items were retained, removing negatively worded items may have increased apparent scale homogeneity. Future research should combine behavioral or multi-source data with independently validated measurement instruments.

Fourth, the study used a convenience sample of self-selected active users and was concentrated among young adults, women, urban respondents, and unmarried or single users, limiting generalizability. Although subgroup analyses and split-sample cross-validation supported the overall pattern, both the exploratory and validation subsamples were drawn from the same convenience sample. Independent replication with broader probability- or quota-based samples is therefore needed.

Fifth, experience-enhancing consumption intention captures willingness to pay rather than actual expenditure or repeated payment behavior. Although respondents with prior paid-purchase experience reported higher willingness to pay, the study did not directly assess transaction records, overspending, or platform-specific payment behavior. Future research should incorporate behavioral payment data to evaluate the consumer consequences of relationship-based AI services more directly.

Sixth, the study did not directly measure psychological well-being, emotional dependence, problematic AI use, or other mental health outcomes. Its public mental health contribution should therefore be interpreted as indirect: the findings identify associations between loneliness and actual-ideal self-discrepancy, relational engagement, and consumption- and continuance-related intentions rather than demonstrating mental health benefits or harms. Future research should incorporate validated mental health and problematic-use measures to examine these implications directly.

### 5.5 Conclusion

This study tested, through a competing-mediation model, how loneliness and actual-ideal self-discrepancy are associated with consumer responses through two theoretically specified pathways: emotional bonding with the AI and escapism. The results consistently indicate that the relational compensation pathway, represented by emotional bonding with the AI, is more strongly associated with experience-enhancing consumption intention and continuance intention than the avoidant compensation pathway represented by escapism. These findings move beyond a view of AI companion use as primarily a form of escape and suggest that relationship-like engagement with AI may be an important correlate of consumption-related and continuance intentions.

These findings should not be interpreted as evidence of direct mental health effects. Rather, they identify a potentially relevant pathway through which higher levels of loneliness and actual-ideal self-discrepancy may be associated with deeper relational engagement with AI companions and with stronger consumption-related and continuance intentions.

## Data Availability

The original contributions presented in the study are included in the article/Supplementary material, further inquiries can be directed to the corresponding author.
