# Supplementary material for: Loneliness to consumption intention: emotional bonding and escapism in AI emotional companion app use
Source: Front Public Health. 2026 Oct 2;14:1964252. doi: 10.3389/fpubh.2026.1964252 (PMC13672010; doi:10.3389/fpubh.2026.1964252)
Supplement: Supplementary file 1 [file Supplementary_file_1.DOCX]

Table S1. Measurement items for the study constructs

| **Item** | **Measurement item** |
| --- | --- |
| LON1 | I often feel lonely. |
| LON2 | I feel disconnected from the people around me. |
| LON3 | I feel that there are too few people who understand me. |
| LON4 | I feel emotionally isolated. |
| LON5 | I feel that I lack people to spend time with. |
| LON6 (R) | I feel sufficiently connected to the people around me. |
| SIG1 | I feel there is a large gap between who I am now and the ideal person I want to become. |
| SIG2 | I feel that my actual self falls short of my ideal self. |
| SIG3 | I feel there is a considerable distance between the life I want and my current life. |
| SIG4 | I feel that my actual self is quite different from the ideal self I aspire to. |
| SIG5 | I want to experience, in some other way, the self I have not been able to realize in reality. |
| SIG6 (R) | On the whole I am satisfied with who I am now, and I feel the gap from my ideal self is small. |
| ANT1 | This AI character feels as though it behaves like a human. |
| ANT2 | This AI character feels as though it has a personality similar to a person’s. |
| ANT3 | This AI character feels like a being that has emotions. |
| ANT4 | This AI character feels like a being with human qualities. |
| ANT5 | This AI character feels closer to a real person than to a mere machine. |
| ANT6 (R) | This AI character feels mechanical and impersonal. |
| ER1 | This AI character feels as though it understands my emotions well. |
| ER2 | This AI character responds appropriately to the emotions I feel. |
| ER3 | This AI character responds sensitively to changes in my emotions. |
| ER4 | This AI character feels as though it empathizes with my emotional state. |
| ER5 | This AI character gives me a feeling of emotional support. |
| ER6 (R) | I feel that this AI character’s responses are always the same, regardless of my emotions. |
| PI1 | I feel as though I have formed an intimate relationship with this AI character. |
| PI2 | I feel that I know this AI character well on a personal level. |
| PI3 | This AI character feels like a friend. |
| PI4 | I feel emotionally connected to this AI character. |
| PI5 | Interaction with this AI character feels like a relationship with a real person. |
| PI6 (R) | This AI character feels unfamiliar to me, and I feel no closeness to it. |
| ESC1 | While using this AI character, I briefly forget about my real-world problems. |
| ESC2 | When interacting with this AI character, I feel as though I have escaped from reality. |
| ESC3 | This AI character lets me get away from daily life for a while. |
| ESC4 | I find myself using this AI character to forget stress or worries. |
| ESC5 | Using this AI character feels like a kind of means of escaping reality. |
| ESC6 (R) | Even when I interact with this AI character, I do not feel any escape from my real-world problems. |
| EECI1 | I am willing to pay to enhance my experience with this AI character. |
| EECI2 | I am interested in paid services for personalizing or customizing this AI character. |
| EECI3 | I am willing to pay for premium features or content in this AI character app. |
| EECI4 | I am willing to purchase upgrade services for richer interaction with this AI character. |
| EECI5 | Overall, my willingness to spend on services related to this AI character is positive. |
| EECI6 (R) | I think spending money on paid services related to this AI character is not worthwhile. |
| CI1 | I intend to keep using this AI character in the future. |
| CI2 | I intend to continue using this AI character. |
| CI3 | I plan to use this AI character frequently. |
| CI4 | I have no intention of stopping my use of this AI character. |
| CI5 (R) | I am likely to stop using this AI character in the near future. |
