# Supplementary material for: Loneliness to consumption intention: emotional bonding and escapism in AI emotional companion app use
Source: Front Public Health. 2026 Oct 2;14:1964252. doi: 10.3389/fpubh.2026.1964252 (PMC13672010; doi:10.3389/fpubh.2026.1964252)
Supplement: Supplementary file 2 [file Supplementary_file_2.DOCX]

Table S2. Robustness comparison of models with and without reverse-coded items

| **Indicator** | **39-item model** | **47-item model** |
| --- | --- | --- |
| Measurement χ²/df | 1.82 | 2.04 |
| Measurement CFI | .950 | .914 |
| Measurement TLI | .946 | .908 |
| Measurement RMSEA | .046 | .051 |
| Structural χ²/df | 2.12 | 2.24 |
| Structural CFI | .930 | .897 |
| Structural TLI | .925 | .891 |
| Structural RMSEA | .054 | .056 |
| Measurement SRMR | .042 | .053 |
| LON → BOND | .177 | .188 |
| SIG → BOND | .391 | .374 |
| LON → ESC | .293 | .313 |
| SIG → ESC | .305 | .284 |
| BOND → EECI | .667 | .660 |
| ESC → EECI | −.020 | −.021 |
| BOND → CI | .807 | .806 |
| ESC → CI | .027 | .021 |

Note. The 47-item model retained all eight reverse-coded items after reverse scoring. The 39-item model corresponds to the primary specification reported in the manuscript.
