# Supplementary material for: Loneliness to consumption intention: emotional bonding and escapism in AI emotional companion app use
Source: Front Public Health. 2026 Oct 2;14:1964252. doi: 10.3389/fpubh.2026.1964252 (PMC13672010; doi:10.3389/fpubh.2026.1964252)
Supplement: Supplementary file 3 [file Supplementary_file_3.DOCX]

Table S3. Split-sample cross-validation of the measurement model

| **Analysis** | **Exploratory subsample** | **Validation subsample** |
| --- | --- | --- |
| N | 196 | 197 |
| Procedure | PAF + Promax | Second-order CFA |
| KMO | .934 | — |
| Bartlett’s χ² | 5931.50*** | — |
| Primary item-loading range | .392-.866 | .531-.906 |
| χ²/df | — | 1.62 |
| CFI | — | .926 |
| TLI | — | .920 |
| RMSEA | — | .056 |
| SRMR | — | .057 |
| BOND→ANT | — | .912 |
| BOND→ER | — | .938 |
| BOND→PI | — | .931 |
