# Supplementary material for: Loneliness to consumption intention: emotional bonding and escapism in AI emotional companion app use
Source: Front Public Health. 2026 Oct 2;14:1964252. doi: 10.3389/fpubh.2026.1964252 (PMC13672010; doi:10.3389/fpubh.2026.1964252)
Supplement: Supplementary file 4 [file Supplementary_file_4.DOCX]

Table S4. CFA-based common latent method-factor assessment

| **Model** | **χ²** | **df** | **CFI** | **TLI** | **RMSEA** |
| --- | --- | --- | --- | --- | --- |
| Correlated-trait CFA | 1,200.51 | 674 | .953 | .948 | .045 |
| Trait + common method factor | 1,044.93 | 635 | .963 | .957 | .041 |
